# HIV Preexposure Prophylaxis in the U.S. Military Services — 2014–2016

**DOI:** 10.15585/mmwr.mm6720a1

**Published:** 2018-05-25

**Authors:** Jason M. Blaylock, Shilpa Hakre, Jason F. Okulicz, Eric Garges, Kerry Wilson, Jenny Lay, Ellen A. Roska, Nelson L. Michael, Charmagne G. Beckett, Steven B. Cersovsky, Sheila A. Peel, Paul T. Scott

**Affiliations:** ^1^Infectious Disease Service, Walter Reed National Military Medical Center, Bethesda, Maryland; ^2^Henry M. Jackson Foundation for the Advancement of Military Medicine, Inc., Bethesda, Maryland; ^3^U.S. Military HIV Research Program, Walter Reed Army Institute of Research, Silver Spring, Maryland; ^4^Infectious Disease Service, San Antonio Military Medical Center, San Antonio, Texas; ^5^Department of Preventive Medicine and Biostatistics, Uniformed Services University of the Health Sciences, Bethesda, Maryland; ^6^Department of Preventive Medicine and Biometrics, Walter Reed Army Institute of Research, Silver Spring, Maryland; ^7^Pharmacy Operations Division, Defense Health Agency, Falls Church, Virginia; ^8^Navy Bloodborne Infection Management Center, Portsmouth, Virginia; ^9^U.S. Army Public Health Center, Aberdeen Proving Ground, Maryland.

Human immunodeficiency virus (HIV) infection is a substantial health concern for the U.S. Department of Defense (DoD) and for service members stationed throughout the world. Each year, approximately 350 new HIV infections are diagnosed in members of the U.S. military services, with most infections acquired within the United States (*1*). The DoD populations most affected by HIV mirror those in the U.S. civilian population; the highest rates of new military diagnoses are in men and blacks or African Americans (blacks) (*1*). Blacks are disproportionally affected, and most new diagnoses occur among men who have sex with men (MSM). HIV preexposure prophylaxis (PrEP) is approximately 90% effective in preventing HIV infection when used properly (*2*), and an increasing number of active duty personnel have used HIV prevention services and PrEP in the military health system since the repeal of “Don’t Ask, Don’t Tell”* in 2011 (*3*). Military health system and service records were reviewed to describe HIV PrEP use among military personnel, and military health care providers were surveyed to assess HIV PrEP knowledge and attitudes. Among 769 service members prescribed PrEP during February 1, 2014–June 10, 2016, 60% received prescriptions from an infectious disease provider, 19% were black men, and 42% were aged >28 years. Half of surveyed military health care providers self-rated their PrEP knowledge as poor. DoD is developing new policy to address access to care challenges by defining requirements and establishing pathways for universal patient access to PrEP.

Charts were reviewed for service members without a diagnosis of HIV infection whose records indicated a prescription for emtricitabine/tenofovir disoproxil fumarate (Truvada, Gilead Sciences, Inc.) during February 1, 2014–June 10, 2016, and data were collected on demographic characteristics, service branch, risk behavior, and MSM risk index (*4*). The MSM risk index is a validated seven-item screening index used to prioritize patients for intensive HIV prevention efforts, including PrEP, with a score ≥10 having a sensitivity and specificity of 84% and 45%, respectively (*5*). Laboratory data were obtained from the Defense Medical Surveillance System (*6*). Infection status was ascertained by negative fourth generation HIV antigen/antibody testing and HIV viral load when clinically indicated. During 2015–2017, surveys were administered to 4,217 primary care and infectious disease providers in the Army, Navy, and Air Force to evaluate knowledge, attitudes, experience, and beliefs related to HIV PrEP.

Among 769 service members without HIV infection who were prescribed Truvada during February 1, 2014–June 10, 2016, 759 (99%) were men, and 320 (42%) were aged >28 years, including 57 aged >40 years (Table) (Figure 1). Blacks accounted for 19% of those prescribed Truvada, compared with 47% who were white. Among the 769 Truvada recipients (including 33 whose education level was unknown), 285 (37%) had at least some college education. The indication for initiating PrEP was most commonly sexual contact with men (87%) and condomless sex (73%); 30% reported exposure to sexual partners with known HIV infection. The MSM risk index score was documented for 156 (20%) PrEP prescription recipients; among those for whom MSM risk index score was available, 72% had scores ≥10.

**TABLE:** Number of U.S. military service members (N = 769) without human immunodeficiency virus (HIV) infection who initiated preexposure prophylaxis, by selected characteristics — February 1, 2014–June 10, 2016

Characteristic	No. (%)
**Total**	**769 (100)**
**Sex**	
Men	759 (99)
Women	10 (1)
**Age group (yrs)**	
18–28	449 (58)
29–40	263 (34)
41–48	44 (6)
≥49	13 (2)
**Race**	
White	361 (47)
Black	149 (19)
Other*	259 (34)
**Service branch**	
Army	207 (27)
Navy	364 (47)
Air Force	158 (21)
Marine Corps	40 (5)
**Education, highest level**	
High school or less	451 (59)
Some college	84 (11)
Bachelor's degree	120 (16)
Higher than bachelor's degree	81 (11)
Unknown	33 (4)

* Includes American Indian/Alaska Native, Asian, Native Hawaiian/Pacific Islander, and unknown.

**FIGURE 1:**
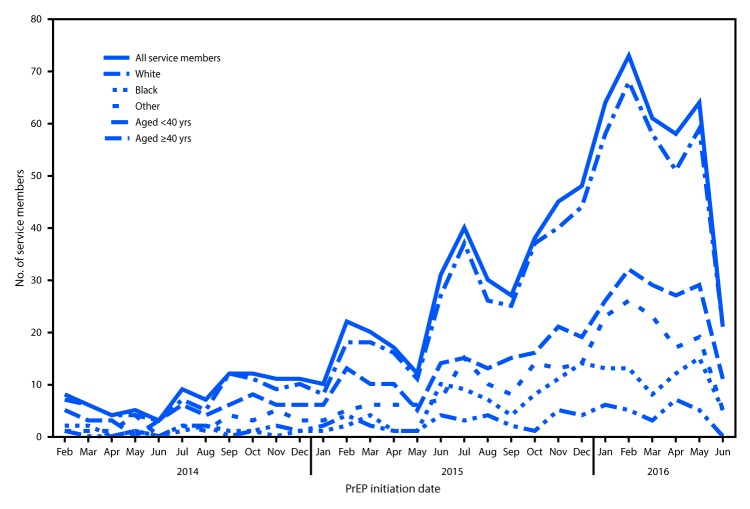
Number of military service members who initiated human immunodeficiency virus (HIV) preexposure prophylaxis (PrEP) among U.S. military personnel on active service who did not have HIV infection, by month — 2014–2016* * Any patient without HIV infection who received an initial prescription for Truvada paid by the U.S. Department of Defense during February 1, 2014–June 10, 2016 was considered to have received HIV PrEP.

Service members who received PrEP were assigned to duty locations throughout the United States and several locations overseas; 315 (41%) of all PrEP recipients accessed services at one of three medical centers located in the Maryland/District of Columbia area; Portsmouth, Virginia; and San Diego, California (Figure 2). Of the 769 Truvada recipients, 464 (60%) accessed PrEP at infectious disease clinics. The majority had appropriate laboratory screening; however, 16% did not have an HIV test within 14 days of initiating PrEP, 13% were never evaluated for hepatitis B virus infection, and 20% and 30% did not have kidney function assessed at baseline or within 90 days of PrEP initiation, contrary to recommendations.

**FIGURE 2:**
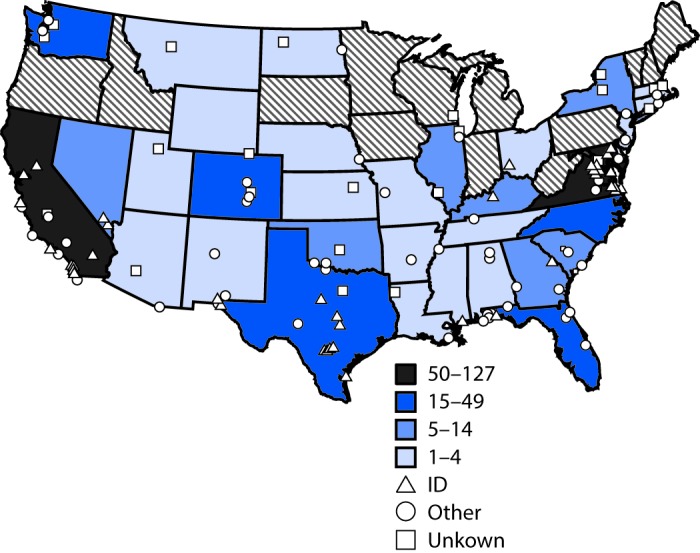
Number of military service members who initiated human immunodeficiency virus (HIV) preexposure prophylaxis (PrEP) among U.S. military personnel on active service who did not have HIV infection, by location of duty and prescribing clinic type — 2014–2016* * Any patient without HIV infection who received an initial prescription for Truvada paid by the U.S. Department of Defense during February 1, 2014–June 10, 2016 was considered to have received HIV PrEP.

Among the 4,217 Army, Navy, and Air Force health care providers who were asked to respond to a web-based survey, 1,599 (38%) responded, including 1,190 (74% of respondents) primary care providers. Overall, 789 (49%) respondents rated their knowledge of PrEP as poor, and 470 (29%) reported ever having prescribed PrEP. Common health care provider concerns included medication adverse effects (915; 57%), compliance (817; 51%), and a need for more clear evidence of safety or efficacy (812; 51%). Despite these limitations and concerns, 1,082 (68%) of the responding health care providers endorsed provision of PrEP in the military health care system.

## Discussion

A key goal of the national HIV prevention strategy is effective use of HIV prevention services, including PrEP.^†^ As in the U.S. civilian population, in the military, HIV disproportionately affects blacks, who represent 17% of the military force^§^ but account for approximately half of all military HIV diagnoses (*7*); during the 2014–2016 study period, only 19% of service members who used PrEP services were black. Further studies are required to learn whether this represents a true disparity and whether improving culturally appropriate efforts will increase PrEP use among black service members who are at increased risk for acquiring HIV infection.

Based on the assumptions that 1) men constitute 85% of the 1.3 million active duty service members, 2) an estimated 4.23% of these men are MSM (including those who self-reported as gay [0.78%], bisexual [2.15%], or other MSM [1.30%]) (*8*), and 3) 25% of MSM have substantially increased risk for HIV (i.e., are candidates for PrEP) (*9*), an estimated 12,000 service members would be eligible for PrEP. However, as of February 2017, approximately 2,000 service members and their beneficiaries had accessed PrEP (Pharmacy Operations Division, Defense Health Agency, unpublished data, 2018). Most patients currently using PrEP are receiving Truvada from major military medical centers after referral to infectious disease specialists. Although a majority of surveyed military health care providers support the use of PrEP for military beneficiaries, increased capacity through provider education and expanded access to the requisite pharmacy and laboratory support services are necessary to meet the anticipated future demand for PrEP and ensure effective delivery of these services in the primary care setting. The transition to use of a fourth-generation HIV immunoassay for HIV screening throughout the DoD has substantially reduced the failure to diagnose acute HIV infection during the “window period” (i.e., the time between exposure to HIV infection and appearance of the first detectable HIV RNA). However, because of variable access to diagnostic tests, some health care providers expressed concern that patients with acute HIV infection might inappropriately be prescribed PrEP instead of antiretroviral treatment because of unrecognized HIV infection.

The maximum estimated annual cost of PrEP to the military health care system is substantial, and new prescriptions for PrEP are expected to continue to rise. Based on the estimate that approximately 12,000 service members would be eligible for PrEP and the current annual cost of Truvada is $12,000 per user,^¶^ the potential maximum annual cost to the military health care system in drug costs alone would exceed $140 million. However, these cost estimates are largely based on assumptions using data from civilian populations and do not account for the lower costs of potential generic prescriptions; further evaluation is needed. In addition, the cost of PrEP services in the DoD can be weighed against the cost savings of preventing HIV infection in the service member; the average lifetime cost of medical care for a person with HIV infection is estimated to be nearly $450,000 (*10*). In addition, indirect costs associated with HIV-infected personnel who are prohibited from combat deployment might have substantial impact on military unit readiness and ability to accomplish specific missions.

Considerations unique to DoD are associated with initiation and maintenance of PrEP services among service members subject to worldwide assignment and deployment. Clinical, pharmacy, and laboratory services are limited in some deployment settings; moreover, access to expedited laboratory testing for HIV infection and the three-site (throat, rectum, and urine) gonorrhea and chlamydia nucleic acid amplification testing (NAAT) recommended by CDC’s 2017 PrEP guidelines for MSM is either unavailable or not easily accessible at many smaller military medical treatment facilities in the United States. In addition, because some pharmacies have insufficient stock of medication for use for PrEP, not every service member or family member who needs Truvada can obtain it. Occupational considerations also exist. Historically, pilots and air crew members on flight status were prohibited from using Truvada and all other antiretrovirals.** To date, only Navy aviation has formally amended its aeromedical waiver guide to allow PrEP use among pilots and air crew.^††^ In addition, adherence to the recommended 3-month follow-up evaluations can be difficult in light of the often unpredictable training and mission schedules. These differences between military policy and clinical practice have the potential to create confusion for both patients and health care providers with regard to implementation of standard PrEP management.

Approximately 28% of PrEP users with documented MSM risk indices had scores <10. The DoD legacy “Don’t Ask, Don’t Tell” policy and reluctance of service members to disclose MSM status might in part explain why only 20% of PrEP users had a documented MSM risk index score and why 28% of those had scores <10. As a result, in the military setting, the risk index alone might not be a reliable discriminator of candidacy for PrEP services. In addition, sexual relations and physical intimacy between unmarried service members, regardless of sex, in the deployed setting has been historically regarded as unprofessional behavior in a combat environment. The currently accepted practice is to discontinue PrEP because Truvada is considered a nondeployable medication in current combat environments.^§§^

The findings in this report are subject to at least three limitations. First, MSM risk index scores were infrequently documented by health care providers, which might have led to candidacy for PrEP services being misclassified. Second, the reported locations of PrEP initiation were based on uneven availability of PrEP services throughout the military health system, which limits generalizability. Finally, the percentage of survey responses from military health care providers was low, which might have led to misrepresentation of provider knowledge of PrEP.

Despite the universal access to care afforded to service members by the military health care system, there is a recognized need to improve and expand access to PrEP for those patients at highest risk for HIV infection. Currently, the availability of PrEP services is heterogeneous, based on the individual patient’s geographic location. If located close to a tertiary care medical center, a patient typically is referred by a primary care provider to an infectious disease specialist to receive PrEP services. To reduce the barrier of requiring a consult to a subspecialty provider, several locations with infectious disease specialists are now allowing patients to self-refer for PrEP evaluations. Patients located closer to smaller military treatment facilities might find it difficult to access PrEP because resources required for PrEP services might be lacking, including three-site gonorrhea and chlamydia NAAT testing and adequate supplies of Truvada at the military pharmacy. In addition, primary care providers with limited knowledge and experience might lack confidence to provide PrEP services. New DoD policy is being developed to address identified gaps through initiatives to improve health care provider education and so ensure universal access to PrEP at the primary care level, and to standardize pharmacy and laboratory service delivery at all military treatment facilities.

**Summary**

**What is already known about this topic?**

Each year, approximately 350 new human immunodeficiency virus (HIV) infections are diagnosed in U.S. military service members, with most diagnoses occurring among men who have sex with men (MSM).

**What is added by this report?**

Among 769 service members prescribed preexposure prophylaxis (PrEP) during February 1, 2014–June 10, 2016, 87% were MSM. In a survey of health care providers, 49% rated their knowledge of PrEP as poor, and 29% reported ever having prescribed PrEP.

**What are the implications for public health practice?**

Strategies for reducing barriers to receipt of HIV prevention and care services include patient self-referrals for PrEP evaluations and development of new health policy to provide universal access to the provider, laboratory, and pharmacy services required for an effective PrEP program.

## Footnotes

* The 1993 Department of Defense policy that prohibited military personnel from discriminating against service members or applicants who did not disclose their homosexual or bisexual sexual orientation, while barring openly gay, lesbian, or bisexual persons from military service.

† White House Office of National AIDS Policy. National HIV/AIDS strategy for the United States: Updated to 2020; 2016 progress report. December 2016. https://www.whitehouse.gov/sites/whitehouse.gov/files/images/nhas-2016-progress-report.pdf.

§ Office of the Deputy Assistant Secretary of Defense for Military Community and Family Policy, Department of Defense. 2015 demographics: profile of the military community. http://download.militaryonesource.mil/12038/MOS/Reports/2015-Demographics-Report.pdf.

¶ National Acquisition Center and U.S. Department of Veteran Affairs. Pharmaceutical Catalog; 2017 https://www.va.gov/nac/Pharma/List?cboContractNumbers=&cboContractorNames=&txtCriteria1=truvada&TxtNDC=&txtPackage=&cboVAClass=&Sort=1&search=Search.
